# Dietary Sugar Research in Preschoolers: Methodological, Genetic, and Cardiometabolic Considerations

**DOI:** 10.31083/j.rcm2409259

**Published:** 2023-09-18

**Authors:** Jessica Yu, Rahbika Ashraf, Anisha Mahajan, Jaimie L. Hogan, Gerarda Darlington, Andrea C. Buchholz, Alison M. Duncan, Jess Haines, David W.L. Ma, on behalf of the Guelph Family Health Study

**Affiliations:** ^1^Department of Human Health and Nutritional Sciences, University of Guelph, Guelph, ON N1G 2W1, Canada; ^2^Department of Mathematics and Statistics, University of Guelph, Guelph, ON N1G 2W1, Canada; ^3^Department of Family Relations and Applied Nutrition, University of Guelph, Guelph, ON N1G 2W1, Canada

**Keywords:** sugar, diet, methodology, dietary assessment, preschool, children, cardiometabolic, genetics, recommendation

## Abstract

Excess dietary sugar intake increases the risk of unhealthy weight gain, an important cardiometabolic risk factor in children. To further our understanding of this relationship, we performed a narrative review using two approaches. First, research examining dietary sugar intake, its associations with cardiometabolic health, impact of genetics on sweet taste perception and intake, and how genetics moderates the association of dietary sugar intake and cardiometabolic risk factors in preschool-aged children 1.5–5 years old is reviewed. Second, methodological considerations for collecting and analyzing dietary intake of sugar, genetic information, and markers of cardiometabolic health among young children are provided. Our key recommendations include the following for researchers: (1) Further longitudinal research on sugar intake and cardiometabolic risk factors is warranted to inform policy decisions and guidelines for healthy eating in preschool-aged children. (2) Consistency in sugar definitions is needed across research studies to aid with comparisons of results. (3) Select dietary collection tools specific to each study’s aim and sugar definition(s). (4) Limit subjectivity of dietary assessment tools as this impacts interpretation of study results. (5) Choose non-invasive biomarkers of cardiometabolic disease until the strengths and limitations of available biomarkers in preschool-aged children are clarified. (6) Select approaches that account for the polygenic nature of cardiometabolic disease such as genome risk scores and genome wide association studies to assess how genetics moderates the relationship between dietary sugar intake and cardiometabolic risk. This review highlights potential recommendations that will support a research environment to help inform policy decisions and healthy eating policies to reduce cardiometabolic risk in young children.

## 1. Introduction 

Excessive dietary sugar intake is known to increase the risk of unhealthy weight gain, poor diet quality, and nutritional inadequacies, as well as cause dental decay in children [1]. Displacement of nutrient-dense foods by sugar-dense foods is of concern [2]. There are also concerns regarding development of asthma, high blood pressure (BP), and lipid abnormalities with increased consumption of sugar in children [3]. In multiple countries including Canada, United States (US), and Germany, sugar intake of young children exceeds recommendations set by their national health authorities [4, 5, 6, 7, 8, 9].

A growing body of research has examined the relationship between dietary sugar intake and cardiometabolic risk (CMR) through dietary and genetic lenses. For example, dietary sugar intake has been associated with increased BP and lipids; and nutrition education discouraging sugar intake has resulted in higher nutrient density in diets of preschool-aged children [5, 10]. Genetics can partially explain dietary intake of sugar, categories of sweet foods and beverages consumed, and CMR in preschool-aged children [11, 12]. In preschool-aged children, single nucleotide polymorphisms (SNP) of the fat mass and obesity related gene (*FTO*) remain the most important genetic contributors to obesity [13]. However, the pathways by which genetics may contribute to sugar intake and moderate the relationship between dietary sugar intake and CMR are not well understood. Furthermore, the use of different methodologies and definitions contributes to potential inconsistencies and differing interpretation of study findings. These differences are especially prominent at the dietary assessment step and are exacerbated by challenges specific to working with very young children. Combined with sparce research, comparison of results becomes challenging.

### Rationale and Methods

The considerable growing body of knowledge and gaps about sugar intake in children and genetics has led to multiple lines of investigations. Importantly, this narrative review focuses on preschool-aged children where there is limited, but growing research highlighting the importance of this age group. This paper provides an overview of the many different areas where dietary sugar intake and genetics independently and in combination influence cardiometabolic health in preschool-aged children. In the first section, we summarize research examining dietary sugar intake, its associations with cardiometabolic health, the impact of genetics on sweet taste perception and intake, and how genetics and select single nucleotide polymorphisms moderate the association of dietary intake and CMR in preschool-aged children (1.5–5 year of age). Given the research evidence is limited for this specific age group, the PubMed, Omni, and Ovid databases were reviewed for literature to date. The following keywords (alone and/or in combination) were used: preschool, children, sugar intake, cardiometabolic, dental caries, genetics, heritability, single nucleotide polymorphism, solute carrier family 2 member 2 (*SLC2A2*) , dopamine receptor D2 (*DRD2*), taste 1 receptor member 2 (*TAS1R2*), taste 1 receptor member 3 (*TAS1R3*), G protein subunit alpha transducin 3 (*GNAT3*), cannabinoid receptor 1 (*CNR1*), proprotein convertase subtilisin/kexin type 1 (*PCSK1*), insulin receptor (*INSR*), insulin receptor substrate 1 (*IRS-1*), and insulin receptor substrate 2 (*IRS-2*). In the second section, methods used to assess dietary sugar intake, genetic information, and markers of cardiometabolic health; as well as important considerations for their use in preschool-aged children are provided. Finally, key methodological recommendations and gaps in knowledge in this field are summarized in the third section. Together, this review highlights the state of the literature pertaining to sugar intake and CMR in preschool-aged children and the methodological, genetic and cardiometabolic considerations crucial for research in this field. Aligning future research with our recommendations will support a robust research body, which can credibly inform healthy eating policies and recommendations designed to reduce cardiometabolic risk in young children.

## 2. Dietary Sugar Intake, Cardiometabolic Risk, and Genetics in Preschool-Aged Children

### 2.1 Added and Free Sugar Definitions and Recommendations

Consistent definitions related to sugar intake are important to develop dietary recommendations and to assess compliance to these recommendations. However, leading health authorities such as the World Health Organization (WHO) and United States Department of Agriculture (USDA) have published recommendations based on different definitions of sugar, i.e., free and added sugars respectively (Table 1, Ref. [8, 14, 15] and Table 2, Ref. [7, 8, 9, 15, 16, 17, 18, 19, 20, 21]). Importantly, the USDA added sugar and WHO free sugar definitions include the same sugars except for those from fruit juice, thus the WHO has a broader definition for free sugars.

**Table 1.** **Sugar definitions**.

Sugar term	Definition	Examples	Organization	Reference
Added sugars	Sugars that are added to foods as an ingredient during preparation, processing, or at the table. Added sugars do not include naturally occurring sugars such as lactose present in milk and fructose present in whole or cut fruit and 100% fruit juice	Sugars, honey, syrups, fruit juice concentrate that is not diluted to single strength	United States Department of Agriculture, 2018	[14]
Free sugars	Include monosaccharides and disaccharides added to foods and beverages by the manufacturer, cook or consumer, and sugars naturally present in honey, syrups, fruit juices and fruit juice concentrates	Sugars, honey, syrups, sugars from fruit juice concentrate and fruit juice	World Health Organization, 2015	[8]
Total sugars	Total sugars include all sugars found in foods and beverages, whether naturally present in intact fruits, vegetables, and milk products or in the form of added and free sugars	Added sugars, free sugars, sugars naturally occurring in cut fruit and milk	Health Canada, 2019	[15]

**Table 2.** **Selected sugar recommendations for preschool-aged children**.

Organization	How is sugar defined?	Reference
World Health Organization, 2015	“WHO guideline recommends adults and children reduce their daily intake of free sugars to less than 10% of their total energy intake (strong recommendation). A further reduction to below 5% or roughly 25 grams (6 teaspoons) per day would provide additional health benefits (conditional recommendation)” —throughout the life span	[8]
Health Canada, 2019	“Free sugars <10% of total energy” – for ≥2 years	[15]
Heart and Stroke Position Statement, 2014	“The Heart and Stroke Foundation recommends that an individual’s total intake of free sugars not exceed 10% of total daily calorie (energy) intake, and ideally less than 5%.”	[16]
Canadian Diabetes Association Position Statement on Sugars, 2016	“Limit intake of free sugars to less than 10% of total daily calorie (energy) intake. This is approximately 50g (12 teaspoons) of free sugars consumption per day based on a 2000-calorie diet”	[17]
Scientific Advisory Committee on Nutrition (UK), 2015	“≤5% of energy from free sugars” – for >2 years	[18]
European Society for Paediatric Gastroenterology, Hepatology and Nutrition, 2017	“Intake of free sugars should be reduced and minimized with a desirable goal of <5% energy intake in children and adolescents ≥2 to 18 years. Intake should be probably even lower in infants and toddlers <2 years”	[19]
Institute of Medicine, 2005	“Although there were insufficient data to set a UL for added sugars, a maximal intake level of 25 percent or less of energy is suggested to prevent the displacement of foods that are major sources of essential micronutrients”	[20]
American Heart Association- Scientific update, 2017	“Committee recommends: 6 tsp/100 kcal/≤25 g added sugars per day for children and no added sugars for children ≤2 years of age”	[9]
Dietary Guideline Advisory Committee (USA), 2015	“Consume less than 10 percent of calories per day from added sugars”	[21]

This table is adapted from: Mahajan, A. *et al*. (2021) ‘Dietary sugar intake among preschool-aged children: a cross-sectional study’ (Supplementary Materials) , *CMAJ Open*, 9(3), pp. E855–E863. doi: 10.9778/cmajo.20200178 [7].

The WHO recommends that free sugar be limited to <10% energy intake (EI) to reduce risk of cardiovascular disease and dental caries and <5% EI for a further reduction of risk of dental caries [8]. The USDA recommends that added sugar be limited to <10% EI each day (~12 teaspoons per day) [22]. Finally, the American Heart Association (AHA) recommends that children consume ≤25 grams (100 kcal or ~6 teaspoons) of added sugar per day [9].

### 2.2 Gaps in Dietary Sugar Definitions and Sugar Guidelines

Inconsistent definitions of sugar exist within the research literature making it difficult to compare values across databases and causing variation in guidelines, data gathered, and how dietary intakes are compared against guidelines [23]. Differences in guidelines and recommendations such as the use of free or added sugars can lead to lack of clarity and differing opinions. Table 2 provides a snapshot of differing recommendations from various national and international organizations [8, 9, 15, 16, 17, 18, 19, 20, 21, 22, 23].

### 2.3 Sugar Intake of Preschool-Aged Children 

Overconsumption of free and added sugars increases the risk of developing chronic diseases such as type 2 diabetes (T2D) and cardiovascular disease in all population groups [8, 16]. Thus, excessive intakes of these sugars is a public health concern for all children [8, 16]. Dietary patterns are well-established as young as 3 years of age and can extend into adulthood [4]. It is known that added sugar intake increases with age and can be significantly higher in older boys [4, 24]. Currently, free and added sugar intake from all food sources in preschool-aged children (<6 years of age) and their association with the development of CMR factors have not been well-researched, although there has been significant attention focused on sugar-sweetened beverage (SSB) intake in children [24]. To our knowledge, studies have examined sugar intake in older children, and do not examine all food sources of free and added sugar intake. Thus, the development of CMR in preschool-aged children needs to be further examined to implement early life interventions and inform policies for this age group. Improved health outcomes in adolescence and adulthood will follow. The next section reviews cross-sectional studies on free and added sugar intake along with the different food sources of sugar that preschool-aged children (inclusion criteria: includes children 1.5–5 years of age) are consuming (Table 3, Ref. [4, 5, 6, 7, 25, 26, 27]).

**Table 3.** **Sugar intake in preschool-aged children**.

Country	Type of study	Participants	Methods used to collect dietary and/or anthropometric data	Primary findings	Reference
Kranz *et al*., 2005 (USA)	Cross-sectional study: The goal was to examine added sugar intakes in preschool-aged children and how these compare with dietary reference intakes.	N = 5437; ages 2 to 5 y.	-Energy sources were divided into 5 categories ≤10% of energy from added sugar; 11–15%; 16-20%; 21–25% and >25%.	-Most children consumed less than 25% of energy from added sugar.	[4]
			-Used 2-day dietary intake data that was collected using the multiple-pass approach.	-Main sources of sugars included: fruit drinks, regular soft drinks and high-fat desserts.	
Jansen *et al*., 2017 (USA)	Secondary data analysis: The goal was to examine longitudinal associations between intake of added sugar (% calories) and sodium in low socioeconomic status preschool-aged children.	N = 524; ages 2.5 to 6 y.	-Used three 24-h dietary recalls at baseline and follow-up completed with trained dieticians.	-Mean percentage of calories from added sugar at follow up was (mean ± std. deviation): 12.9 ± 5.9 (boys) and 12.8 ± 6.3 (girls).	[6]
				-Main food source of added sugars included: soda.	
Foterek *et al*., 2016 (Germany)	Cross-sectional study: The goal of this study was to determine cross-sectional associations between commercial complementary food and added sugar intake in infancy and prospective relation to added sugar intake to added sugar intake in preschool & primary school-aged children.	N = 288; infancy, preschool-aged, primary school-aged children	-3 consecutive days weighted food records were completed at each time point: 0.5 and 0.75 y (infancy); 3 and 4 y (preschool); 6 and 7 y (primary school).	-In infancy, the majority of the added sugar came from commercial foods.	[5]
		Data collected from: Dortmund	-Trained dietitians visited the enrolled families at home to check for thoroughness of the completed food records.	-In both preschool- and primary-aged children, median added sugar intakes exceeded the World Health Organization (WHO) recommendations for percent total energy. Main sources of added sugar included: sweets and milk products.	
		Nutritional and Anthropometric Longitudinally Designed			
		(DONALD) Study.			
Ramsay *et al*., 2018 (USA)	Cross-sectional study: To investigate the differences in the nutritional intake and food consumption between children who consume breakfast versus those that do not.	Dietary data reviewed for 2 y to 5 y (n = 3443) and 6 y to 12 y (n = 5147) from NHANES 2005–2012.	-24-h dietary recall using the Automated Multiple Pass approach.	- For the ages 2 to 5 y it was noted that 4% of the children skipped breakfast while for the ages 6 to 12 y, 14% of the children skipped breakfast.	[25]
			-Categories were constructed for participants that ate or skipped breakfast as reported by the parent.	-Children who skipped breakfast had 40% of day’s intake from snacks (2332 kJ out of 5911 kJ) and high energy as added sugars within the snacks (586 kJ).	
				-Snacks and sweets were one of the leading sources of energy when breakfast was skipped.	
Crowe *et al*., 2020 (Ireland)	-Secondary data analysis: 2 national surveys in Ireland: Growing up in Ireland (GUI) and National Preschool Nutrition Survey (NPNS). The aim was to quantify the total sugar and free sugar intakes for preschool-aged children.	GUI = N = 9793; NPNS = N = 126; age = 3 y.	-Utilized semi-weighted food diaries and short food questionnaires.	-Free Sugar = 40 ± 23.5 g/day and contributed to (mean and std. dev) 14.1% ± 5.81 total energy intake; 75% of the 3 y old children consumed more than 10% total energy intake as free sugar	[26]
			-Data mapping for matching covered and non-covered GUI and NPNS food data.	-Main food sources of free sugar included: fruit juice and smoothies, dairy products, confectionary and soft drinks.	
Devenish *et al*., 2019 (Australia)	-Participants recruited from the SMILE cohort study. The goal of this study was to determine free sugar intakes, sources and determinants of high intakes in preschool-aged children.	N = 938 participants were 2 y old.	-Food frequency questionnaire contained 89 items and were semi-quantitative. These were emailed or posted to parents when their child reached 2 y.	-Mean intake of free sugar was 29.3g/day providing a total of 10% of estimated energy requirements (EER). 71.1% of the children exceeded 5% of their EER and 38.4% of children exceeded 10% of their EER	[27]
				-Main food sources of free sugar intake -cereal-based products and dishes, non-alcoholic beverages, milk products and dishes, infant formula and foods, sugar products and dishes and confectionery and cereal nut/fruit/seed bars.	
Mahajan *et al*., 2021 (Canada)	Cross-sectional study. The aim of this study was to quantify the amount of total, free and added sugar intakes and to examine associations between total, free and added sugar with anthropometric measures (body weight, waist circumference, BMI Z-scores and percent fat mass).	N = 109; ages 1.5 to 5 y	-Used 3-day food records for each participant reviewed by trained staff.	-80% of the children are currently exceeding free sugar intake above the 5% of total energy intake recommendation by the World Health Organization.	[7]
			-Trained staff completed anthropometric measurements for all children.	-Main food source for free and added sugars included: bakery products.	

One study assessed the diets of preschool-aged children (n = 5437) between 2 to 5 years old from the National Health and Nutrition Examination Survey (NHANES) (1988–1992). This study collected two non-consecutive 24-hour recalls from children and found that 11% of participants (2 to 3 years) and 12% of participants (4 to 5 years) exceeded 25% EI per day from added sugar [4]. Furthermore, 72% of participants aged 2 to 3 years and 79% of those aged 4 to 5 years, exceeded 10% EI per day from added sugar [4]. Intake of added sugars in this population exceeded recommendations from the Institute of Medicine and the WHO [4]. Additionally, the primary sources of added sugar in the children’s diets were fruit drinks, high-fat desserts (such as ice cream, pies, cookies, cakes), and regular soft drinks [4]. Furthermore, as added sugar levels increased in the dietary pattern, intake of all other macro and micronutrients (except for carbohydrates and vitamin C) decreased. Finally, children with added sugar intake >25% EI had lower intakes of fibre, protein and fat and higher intake of carbohydrates [4].

Similar findings of preschoolers exceeding sugar recommendations have also been observed in the Growing Healthy Study, an obesity prevention trial in the US [6]. This secondary data analysis included n = 524 preschool-aged children of low socioeconomic status, with a mean ± SD age of 4.1 ± 0.5 years [6]. Three 24-hour diet recalls were completed, including two weekdays and one weekend day and diet-related study measures included %EI from added sugar. Results showed that the mean added sugar intake (mean ± SD) was 12.9 ± 5.9 % EI per day for boys and 12.8 ± 6.3% EI per day for girls. Finally, similar to the previous study [4], a significant proportion of children exceeded WHO recommendations with 69% of boys and 65% of girls exceeding 10% EI per day from added sugars [6].

The Dortmund Nutritional and Anthropometric Longitudinally Designed Study in Germany (n = 288 participants) examined added sugar intake in infancy (through commercial complementary foods) and its relationship with added sugar intake in later years, i.e., preschool-aged (3 to 4 years) and primary school-aged (6 to 7 years) [5]. For each participant, a weighed food record was used to assess diets from three consecutive days and %EI per day was determined. Mean added sugar was reported to be 34.2 g per day and 11.9 %EI per day for preschool-aged children, and 49.7 g per day and 13.4% EI per day for primary school-aged children [5]. It was concluded that a higher intake of added sugar from commercial foods in infancy may predispose children to higher added sugar intakes in preschool-aged (*p* < 0.041) and primary school-aged children (*p* < 0.039). Preschool- and primary-aged children in this population exceeded the recommended intake of added sugar by the WHO and the American Heart Association (AHA) [5].

The effect of skipping the first meal, breakfast, on daily sugar intake has also been examined through a cross-sectional analysis of participants (n = 3443), 2 to 5 years old from the US NHANES 2005-2012 cohort [25]. Two 24-hour diet recalls were used to determine daily added sugar intake and its relationship with breakfast consumption. Mean added sugar intake for preschool-aged children who skipped breakfast was 62 g/day [25] and lower for those who did not skip breakfast, only 51 g/day [25]. It was concluded that preschool-aged children in this population exceeded the recommended intake of added sugar from the AHA regardless of breakfast consumption, but those who skipped breakfast had an even higher intake of added sugar.

Examination of food sources of free sugar has revealed important trends and insights into sugar intake in children. An Irish study of free sugar intake in 3-year-old children including two national data sets (Growing Up in Ireland n = 9793, 51% male; National Preschool Nutrition Survey n = 126, 48% male) [26] found that 75% of the preschool-aged children had free sugar above the <10% EI recommended by the WHO and less than 4% of the study sample attained the <5% EI WHO recommendation. It was noted that the median frequency of free sugar consumption was 4.0 (range of 3.0–5.0) times per day. The main food sources of free sugar were fruit juice and smoothies, dairy products, confectionery, and soft drinks [26].

The SMILE study, using dietary data from 2-year old Australian children (n = 1043) found that 71% and 38% of the children exceeded the WHO recommendations of <5% EI and <10% EI from free sugars, respectively [27]. This study found the primary food sources of free sugar were fruit juices, biscuits, cakes, desserts; and confectionery, with yogurt and non-dairy milk alternatives [27]. In a Canadian study of preschool-aged children, the Guelph Family Health Study found that 80% of 109 preschool-aged participants (aged 1.5–5 years) had free sugar intake above the <5% EI daily recommendation set by the WHO [7]. The most common food sources of free and added sugar were bakery products, sugars and sweets, and cereals and grain products [7].

Overall, these cross-sectional studies across multiple countries reveal that young children exceed global and national recommendations in different countries for free and added sugar intake and are consuming sugars from a variety of food sources.

### 2.4 Sugars from Sugar-Sweetened Beverages and Cardiometabolic Health in Preschool-Aged Children 

The following section describes cross-sectional and longitudinal studies investigating the associations between sugars from SSBs and CMR factors among preschool-aged children (Table 4, Ref. [28, 29, 30, 31, 32, 33, 34, 35]).

**Table 4.** **Sugar intake associations with cardiometabolic risk factors in preschool-aged children**.

Authors	Type of study	Participants	Methods used to collect dietary and/or anthropometric data	Primary findings	Reference
Eny *et al*., 2020 (Canada)	Cohort study- TARGet Kids! repeated measures study examining sugar sweetened beverage (SSB) consumption (including 100% fruit juice)	-Children under 6 y recruited between 2008 to 2017 during well child physician visits from 11 primary care practices.	-Canadian community health survey included a question on frequency of consumption, i.e., “how many drinks of each drink your child has in a typical day in cups?”	-Higher SSB intake was associated with higher cardiometabolic risk score, lower high density lipoprotein cholesterol (HDL-c) and higher triglycerides (TG).	[28]
	and cardiometabolic risk outcomes in preschool-aged children (2008–2017).	-The final analytic sample N = 1778 children between 3 to 6 y.	-Trained research staff measured systolic blood pressure and waist circumference (WC), high density lipoprotein cholesterol, triglycerides and glucose were taken. The following anthropometric measures were completed: child’s height and weight; body mass index (BMI) Z-scores calculated; and child’s birthweight were reported by their parents.	-100% fruit juice and SSB intake was associated with lower HDL-c.	
				-For every 1 cup increased SSB intake the cardiometabolic risk score increased by 0.05 SD units.	
Kosova *et al*., 2013 (USA)	Cross-sectional analysis of US NHANES data (1994 to 2004). The aim of this study was to assess the association between SSB intake and cardiometabolic risk markers in young children.	N = 4880; Ages: 3 to 11 y.	-Home interviews were completed by trained staff. In addition, participants were asked to visit the examination centre to complete a physical examination, provide blood and urine samples and complete questionnaires.	-Overall results for 3 to 11 y- Increased SSB was independently associated with increased CRP concentrations (*p* = 0.003), increased WC (*p* = 0.04), low HDL-c (*p* < 0.001)	[29]
			-Outcome variables: Total cholesterol, HDL-c, low density lipoprotein cholesterol (LDL-c), TG, C-reactive protein (CRP), waist circumference (WC), body mass index (BMI) percentile for age-sex.	- When a subgroup analysis was completed for the following age groups: 3 to 5 y and 6 to 8 y- no associations reached significance except for a positive association between SSB and LDL-c cholesterol levels in the 3 to 5 y age group.	
de Boer *et al*., 2018 (The Netherlands)	Cross-sectional study data collected in 2008–2010. The study aim included to examine the associations between SSB intake, blood pressure and autonomic nervous system in young children.	Ages 5 to 6 y (N = 2519) or 2015–2016 at age 11 to 12 y (N = 769).	-SSB intake at 5 to 6 y of age was reported by the main caregiver	-For the 5 to 6 y age group: Consumed on an average 2.6 SSB servings daily. No associations were found between SSB and blood pressure (BP) after full adjustment of covariates.	[30]
			-SSB intake at 11 to 12 y of age was self-reported by the child	11 and 12 y: Consumed on an average 4.4 SSB servings daily. For every one consumption of SSB serving increase/day - this was associated with a 0.8 mmHg increase in SBP; 0.3 mmHg increase in DBP and 0.9 msec decrease in pre-ejection period.	
			-Systolic blood pressure (SBP), diastolic blood pressure (DBP) and autonomic nervous system were measured in supine position.		
DeBoer *et al*., 2013 (USA)	Longitudinal and cross-sectional study to examine association of SSB intake with weight status for children aged 2 to 5 y.	N = 9600 children in the Early Childhood Longitudinal Survey Birth Cohort.	-Parents were interviewed by trained staff to review children’s SSB intake	-SSB consumption was low at 2 y of age and increased at 4 and 5 y of age.	[31]
			-Trained staff measured height and weight of children	-The study demonstrated that those children that consumed SSB compared to those that didn’t had a higher BMI Z-scores, i.e., ≥1 SSB serving daily at 2 years had a greater increase in BMI Z-scores at 4 y of age.	
			-These evaluations were completed at 2, 4 and 5 y of age	-By age 5 y regular SSB drinkers had higher adjusted odds of obesity (1.43; *p* < 0.01)	
Leermakers *et al*., 2015 (The Netherlands)	Longitudinal cohort study. The aim of this study was to examine the association with SSB intake at 13 months with body composition at 6 y and BMI changes until 6 y.	N = 2045 Dutch children with median age of 5.9 y.	-Semi quantitative food frequency questionnaire (FFQ) (211-item) was completed by parents at age 13 mo.	-For boys, higher intake of SSB was associated with higher BMI scores at 2, 3, 4 and 6 y of age. For girls, there was no association noted between SSB intake and BMI.	[32]
			-Trained staff measured children’s height and weight at the research centres and gathered this data. information from 13 mo to 48 mo.	-Overall, there was no association of SSB intake with body fat percentage in all children.	
			- The children visited the research centre at age 6 y - body fat was measured using dual x-ray absorptiometry (DXA) scans. Percent body fat mass was calculated.		
Dubois *et al*., 2007 (Canada)	Longitudinal Study of Child development in Quebec (1998 to 2002). The goal of this study was to investigate the relationship between SSB intake between meals at 2.5 y, 3.5 y and 4.5 y with prevalence of overweight status in preschool-aged children.	N = 2103 children born in 1998 in Quebec, Canada and n = 1944 children of 4 to 5 y that participated in the study.	-Self-administered FFQ completed by children’s mother when children were aged at 2.5 y, 3.5 y and 4.5 y and a 24 h recall was completed by the research team for children at 4.5 y. This information helped to determine frequency of SSB intake in between meals.	-Regular SSB intake between meals can lead to young children being at greater risk of being overweight.	[33]
			-Children’s height and weight were recorded at 2.5 y, 3.5 y and 4.5 y	-Children that were regularly consuming SSB (versus those not consuming SSB regularly), at 2.5 y, 3.5 y and 4.5 y were overweight at 4.5 y (15% overweight for regular SSB consumers vs. 7% overweight for non SSB consumers).	
Pan *et al*., 2014 (USA)	Longitudinal cohort study- goal was to examine association of SSB intake at infancy with obesity at 6 y of age.	N = 1189 American children that were recruited for the Infant feeding practices Study II (2005–2007) were followed from 10 to 12 mo up to 6 y of age.	-Questionnaires were mailed out in infancy q monthly until 12 mo of age that included the question on frequency of SSB consumed by children.	-Prevalence of obesity at 6 y among children that consumed SSB was 17%, i.e., was 2 times higher than non SSB consumers.	[34]
			-Children’s mother’s measured height and weight of their children at age 6 y.	-If SSB was introduced in infancy, the odds of obesity at 6 y of age was 71% higher for children with SSB intake versus no SSB intake.	
Herbst *et al*., 2011 (Germany)	Longitudinal Study. This study evaluated associations between added sugar intake in early childhood and BMI/body fat at 7 y of age.	N = 216 (111 boys; 105 girls) recruited in the Dortmund Nutritional and Anthropometric Longitudinally Designed Study	-3 consecutive days weighted food records were completed	-At 1 y of age- 4% of daily energy intake came from total added sugar; between 1 and 2 y of age, the total added sugar intake increased by 4% of energy intake.	[35]
		-Added sugar intake was plotted at 0.5, 1.5, 2 y of age and then BMI and % body fat was recorded at 7 y of age	-Trained dietitians visited the enrolled families at home to check for thoroughness of the completed food records.	-Common added sugar intake sources included: dairy products, sweet spreads, cakes and pastries and sweets.	
				-At 1 y of age, higher total added sugar was related to lower BMI at 7 y of age	
				-Between ages 1 to 2 y, increases in added sugar intake led to an increase in BMI at 7 y of age	
				-No associations noted for % body fat.	

A cross-sectional study linking SSB intake with CMR factors in n = 1778 participants between the ages of 3 to 6 years [28] highlighted that SSB consumption was positively associated with CMR scores, specifically lower high-density lipoprotein (HDL-c) and higher triglycerides (TG). This study utilized the CMR scores as described by the American Academy of Pediatrics and included systolic blood pressure, TG, waist circumference (WC), glucose, and HDL-c [28]. The authors demonstrated that with every 1 cup of SSB intake, there was a significant increase in the CMR score by 0.05 SD units [28]. It was also noted that 100% fruit juice did not have a significant association with the CMR factors except lower HDL-c was reported [28].

Similarly, cross-sectional analyses of NHANES (1994 to 2004) data including children (n = 4880) aged 3 to 11 years found that the highest tertile of SSB consumption (4.39 ± 1.71 SSB servings (or 35 oz daily)) was positively associated with the C- reactive protein (CRP) and WC and negatively associated with HDL-c [29]. A subgroup analysis of children aged 3 to 5 years found a positive association between SSB intake and low density lipoprotein cholesterol (LDL-c) [9]. In 3- to 11-year-old children, SSB intake was positively associated with CRP in both sexes; and inversely associated with HDL-c and positively associated with LDL-c in girls [29]. Furthermore, in both non-Hispanic Black and White groups, a significant inverse association was noted between SSB intake and HDL-c. In the non-Hispanic Black group, SSB intake was significantly positively associated with TG and CRP levels [29].

A follow-up study conducted in Amsterdam, further highlighted the longitudinal associations between SSB intake and BP in children aged 5 to 6 years or 11 to 12 years [30]. The study found an average intake of 2.6 servings of SSB per day. There were no associations found between SSB and BP for children aged 5 to 6 years [21]. However, as the number of servings increased in older children (11 to 12 years old), the association between SSB and BP became positive and significant [30].

Similar results were seen in the Early Childhood Longitudinal Birth-Cohort study in the US, where SSB intake in children aged 2 to 5 years (n = 9600) were examined [31]. Study participants with higher SSB intake had higher BMI Z-scores at age 4 years and 5 years but not at 2 years [31]. By 5 years of age, regular SSB drinkers had increased odds of obesity [31]. Another longitudinal study investigating Dutch children with a median age of 5.9 years (n = 2045) found that high SSB consumption in boys was significantly associated with CMR at age 6 years [32]. In yet another longitudinal study from Québec, Canada, children ages 2.5 to 3.5 years (n = 2103) who consumed higher SSBs had more than three times the odds of being overweight at 4.5 years [33].

Studies examining SSB intake over time from infancy onward have revealed similar outcomes. For example, a longitudinal study of SSB intake in children (n = 1189) in the US from infancy to 6 years of age showed that infants exposed to SSBs were twice as likely to be obese compared with infants who were not [34]. Furthermore, the likelihood of consuming SSBs at age 6 years was 71% higher for children who consumed SSBs at infancy and 92% higher for those children who consumed SSBs before 6 months of age [34]. Another study found that excess intake of added sugars (from dairy products, baked goods, sweets and spreads) consumed in the first two years of life were associated with an increased BMI at age 7 years in a subset of the longitudinal Dortmund Nutritional and Anthropometric Longitudinally Designed study (n = 216; 51% male) [35].

Thus, these cross-sectional and longitudinal studies suggest that excessive SSB intake starting from the first few years of life can predispose preschool-aged children towards higher CMR. The next section will discuss genetic research investigating dietary sugar intake, CMR, and their relationship in preschool-aged children.

### 2.5 Genetic Research: Dietary Sugar Intake and Cardiometabolic Risk in Preschool-Aged Children

Dietary sugar intake, genetic variation, and CMR have complex interactions. Studies investigating heritability suggest that genetics plays an important role in dietary sugar intake and CMR in preschool-aged children. However, how genetic variation and gene-diet interactions may impact dietary sugar intake and moderate the association between dietary sugar intake and CMR phenotypes is not well understood in this population.

### 2.6 Heritability of Dietary Sugar Intake

Heritability is defined as the proportion of a traits’ variability that can be attributed to genetic variation. Twin and adoption studies provide estimates of the proportion of variability in a trait that can be attributed to heritability, i.e., additive genetic factors; common environmental factors shared by family members, and unique environmental factors. Heritability of dietary sugar intake is not well studied in adults and even more rare in preschool-aged children. One study investigated 1216 twin pairs born in England and Wales in 2007 [11]. Dietary data collected at a mean age of 21 months revealed that the shared environment predicted 66%, 84%, 91%, and 97% of the variation in dietary intake of milk-based desserts, sweet cereal products, added sugar and confectionary, and juice, respectively. Conversely, genetic factors were estimated to predict 15%, 5%, 5%, and 1% of the same food groups, respectively. These data indicate that a small but significant proportion of dietary sugar intake can be attributed to genetic influences in very young children. However, it is unknown how the proportion of dietary sugar intake attributable to shared environment, genetic factors, and nonshared environment, may change with age.

### 2.7 Heritability of Cardiometabolic Risk 

Twin and adoption studies in adults have established that many components of CMR are highly heritable. One twin study using variable-aged cohorts (age 13–92 years old) from Sweden, Australia, and the Netherlands found that between 48% to 83% of lipid levels, i.e., LDL-c, HDL-c and triglycerides, are heritable [36]. However, twin studies investigating heritability of CMR in children, and how this relates with heritability estimates in adults are rare. In one systematic review of twin and adoption studies of children (largely Caucasian, aged 1 to 18 years old), BMI was found to have moderate to high heritability [12]. This heritability was high in children 1.5 years of age and decreased by approximately age 5 years, before increasing during adolescence. This suggests that environmental factors further influence CMR at different life stages.

### 2.8 Single Nucleotide Polymorphism (SNP) Variation and Tracking Childhood Cardiometabolic Risk into Adulthood 

In adults, SNP variation has only been able to explain a limited amount, i.e., 1.5%–12% of the CMR variation predicted by twin and family studies, and data are even more sparse in children [37]. In 1,169 children from the Tracking Adolescents’ Individual Lives Survey Cohort at 7 years of age, a genetic risk score calculated from 25 genome wide significant SNPs explained 3.6% of the variance of childhood BMI [13]. This cohort was part of a larger genome wide association study (GWAS) meta-analysis of 61,111 children between the ages of 2 and 10 years, which found that genetic background of childhood BMI overlaps with that of adult anthropometric and CMR factors, i.e., BMI, waist-to-hip-ratio, body fat percentage diastolic BP, and T2D [13]. Thus, observed correlations between childhood obesity and adult CMR may be partially explained by shared genetics but may also be explained by strong tracking of childhood BMI into adulthood.

### 2.9 Monogenic Obesity and Hyperglycemia

In children, the most important single gene mutations linked to monogenic obesity include those in the leptin receptor (*LEPR*), melanocortin 4 receptor (*MC4R*), pro-opiomelanocortin (*POMC*) and prohormone convertase 1 (*PCSK1*) genes [38]. Glucokinase Maturity Onset Diabetes of the Young is characterized by juvenile onset hyperglycemia caused by a mutation in the glucokinase gene [39]. The majority of obesity and hyperglycemia cases are polygenic in nature. Monogenic forms of obesity have been found to account for less than 5% of all severe obesity cases and has not been quantified in children [40]. Monogenic forms of hyperglycemia account for 1% to 4% of pediatric diabetes cases [41, 42].

### 2.10 Fat Mass and Obesity Associated gene (FTO)

The *FTO* is the most well-known gene influencing obesity in children and adults. *FTO* polymorphisms that have been associated with overweight/obesity and BMI in preschool aged children include rs9939609 and rs17817449 [13, 43]. In adults, rs55872725 was significantly associated with SSB consumption [44]. Further investigation may reveal more pathways by which *FTO*, sugar intake, and CMR are linked.

### 2.11 Selected Genes Involved in Sugar-Related Pathways and their Relationship with Dietary Sugar Intake and CMR in Preschool-Aged Children

CMR is complex and while key genes have been identified, it is also important to consider potential additive effects across a range of processes that may contribute to overall risk. Since more than 93% of obesity cases have complex polygenic causes, investigations into common genetic risk loci are warranted [40]. This section will discuss SNP variation in sugar-related pathways that have been investigated for associations with dietary intake of sugar and/or CMR factors in preschool-aged children.

**Fig. 1.**
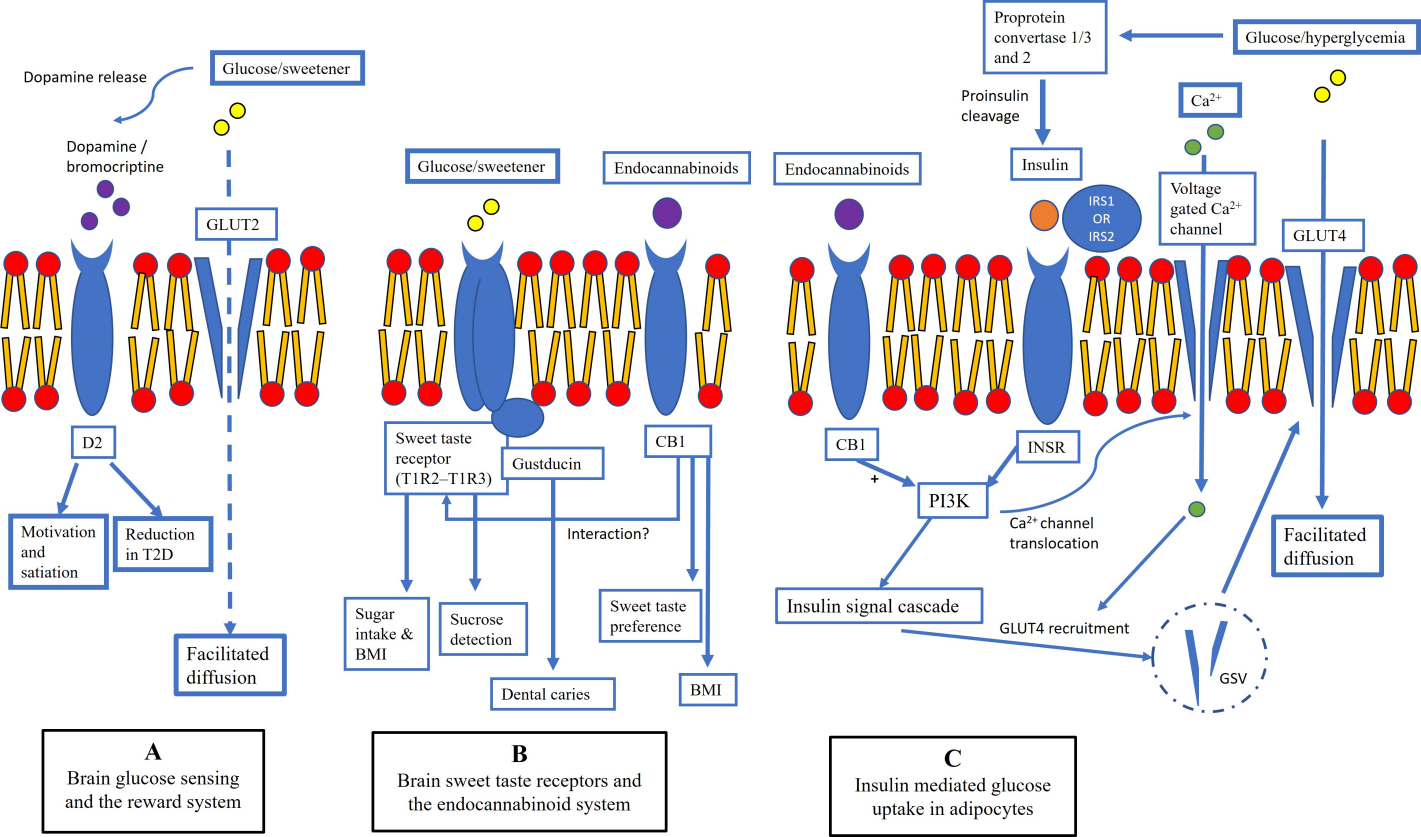
**Genes involved in glucose sensing, the reward system, sweet taste receptors, the endocannabinoid system, and insulin mediated glucose uptake found to have SNP associations with dietary sugar intake and/or cardiometabolic risk factors in children**. (A) The solute carrier family 2 (*SLC2A2*) gene encodes GLUT2†, a facilitated glucose transporter, which has both transport and receptor functions. *DRD2*†encodes dopamine receptor D2. (B) TAS1R2* and TAS1R3* code for proteins (T1R2 and T1R3) that heterodimerize to form sweet taste receptors. GNAT3* encodes the alpha subunit of gustducin, the G protein responsible for transmitting sweet, bitter and umami tastes from taste receptors. Endocannabinoids bind with G-protein coupled cannabinoid receptors, i.e., CB1 and CB2 encoded by *CNR1*†and *CNR2* respectively. (C) Proprotein convertase subtilisin/kexin type 1 and 2 (PCSK1*¤, *PCSK2*¤) encode proprotein convertase 1/3 and 2 (PC1/3, PC2) respectively. INSR*¤ encodes preprotein precursors cleaved into alpha and beta subunits of the heterotetrameric insulin receptor. Binding of ligands including insulin and insulin like growth factor 1 (IGF-1) along with insulin receptor substrate 1 (IRS1¤) or insulin receptor substrate 2 (IRS2) to the insulin receptor results in activation of the insulin signaling pathway. *Genes that have been studied in preschool-aged children (includes 18 months–5 years) for a relationship with dietary sugar intake and/or cardiometabolic risk [45, 46, 47, 48, 49, 50, 51, 52]. †Genes that have been studied in children aged 6–13 years old for a relationship between SNPs with dietary sugar intake and/or cardiometabolic risk and are potentially of interest for studies in preschool-aged children [53, 54, 55, 56]. ¤Genes that have been studied in children aged 4–18 years old for a relationship between SNPs with dietary sugar intake and/or cardiometabolic risk and are potentially of interest for studies in preschool-aged children [57, 58, 59, 60, 61]. BMI, body mass index; CB1, cannabinoid receptor type 1; CB2, cannabinoid receptor type 2; *CNR1*, cannabinoid receptor 1; *CNR2*, cannabinoid receptor 2; *DRD2*, dopamine receptor D2; GLUT2, glucose transporter 2; GLUT4, glucose transporter 4; *GNAT3*, G protein subunit alpha transducin 3; GSV, GLUT4 storage vesicle; INSR, insulin receptor; IRS1, insulin receptor substrate 1; IRS2, insulin receptor substrate; *SLC2A2*, solute carrier family 2 member 2, SNP, single nucleotide polymorphism; *TAS1R2*, taste 1 receptor member 2; *TAS1R3*, taste 1 receptor member 3; T1R2, taste receptor type 1 member 2; T1R3, taste receptor type 1 member 3; *PCSK1*, proprotein convertase subtilisin/kexin type 1; *PCSK2*, proprotein convertase subtilisin/kexin type 2; PC1/3, proprotein convertase 1/3; PC2, proprotein convertase 2; PI3K, phosphatidylinositol 3-kinase; T2D, type 2 diabetes; D2, opamine receptor D2.

In particular, the following selected genes involved in glucose sensing (*SLC2A2*), the reward system (*DRD2*), sweet taste receptors (*TAS1R2*, *TAS1R3*, *GNAT3*), the endocannabinoid system (*CNR1*), and insulin mediated glucose uptake (*PCSK1*,* INSR, IRS-1*, *IRS-2*) are reviewed for potential relationship with sugar intake and CMR. Fig. 1 [45, 46, 47, 48, 49, 50, 51, 52, 53, 54, 55, 56, 57, 58, 59, 60, 61] provides background information of these genes, which have been investigated in young children and may be of interest for future research. Furthermore, studies investigating the relationship between select genetic polymorphisms, dietary sugar intake, and cardiometabolic risk factors in preschool-aged children (1.5–5 years) are reviewed in Table 5 (Ref. [45, 46, 47, 48, 49, 50, 51, 52]). The dietary sugar outcomes reviewed included sugar preference and consumption as well as the presence of dental caries. The CMR outcomes reviewed included BMI Z-scores, overweight/obesity, and TG.

**Table 5.** **Polymorphisms associated with dietary intake and cardiometabolic risk factors in preschool aged children (Inclusion criteria: 18 months–5 years old, up to 12 years)**.

Gene	SNP type	Polymorphism	SNP ID	Outcome	Significant association?	Reference
*TAS1R2*	Non-synonymous	T>C Ile191Val	rs35874116	In 47, mostly Caucasian (87.5%) preschool-aged children (3.47 ± 1.15 years), individuals with the T/T genotype preferred sweet snacks, i.e., consumed higher % calories from sugar and were more likely to consume evening sugary snacks compared to C allele carriers	Y	[45]
	Non-synonymous	T>C Ile191Val	rs35874116	-In 312 Brazilian children (1–7.7 y), Ile carriers consumed more sugar and sugar-dense foods than children with a Val/Val genotype at 3.9 years	Y	[46]
				-Greater weight gain and trend towards higher BMI z-score in Val/Val homozygotes was observed compared to Ile carriers at 3.9 and 7.7 years old.		
*TAS1R3*	Promoter -1266 kb upstream	T>C	rs35744813	- In 101 mixed race children (Mean age (SEM), range) = (7.8 (0.2), 5–10 y) from Pennsylvania, USA, no associations between genotype (CC, CT, TT) and sucrose preference were found.	N	[49]
				-Age × genotype interaction: (CC VS CT + TT)		
				-Mothers but not children preferred lower concentrations of sucrose.		
		T>C	rs35744813	In 84 children (mean age ± SD, range: 8.0 ± 1.9, 5–10 y) from the city of Philadelphia (29 related), the TT genotype was associated with a poorer ability to detect low concentrations of sucrose compared with the C genotype.	Y	[50]
		T>C	rs35744813	In 312 Brazilian children (1–7.7 y), no association with food intake or nutritional status at 1, 3.9 and 7.7 y old was found.	N	[46]
*GNAT3*	Unknown	T>C	rs704871	In 1305 white US children (3–12 y) from the Center for Oral Health Research in Appalachia (COHRA), genotype was nominally associated with dental caries, a result replicated in 1695 white Danish children (2–7 y) from the Denmark National Birth Cohort (*p* = 0.004).	Y	[47]
	Intronic	G>A	rs2074674	In 486 Caucasian children (mean age ± SD, 3.4 ± 1.5 y) from the family based	N	[51]
		T>G	rs6962693	oral health studies of COHRA neither SNP was associated with total caries experience in the primary dentition group		
*PCSK1*	Non-synonymous	Asn221Gln	rs6232:G	-In 1206 unrelated Mexican-Mestizo children (5–12 y), no risk genotype frequency differences were found between lean and overweight children	N	[52]
		Ser690Thr	rs6235:C			
				-Neither rs6232:G or rs6235:C was associated with obesity		
*INSR*	Intronic	G>A	rs7248104	In 544 children from Hamilton, Canada from the Family Atherosclerosis Monitoring in earLY life birth cohort (91.1% European Ancestry), rs7248104 was statistically nominally associated with triglycerides at 5 years before Bonferroni adjustment, but not at 0 or 3 y.	Y	[48]

BMI, body mass index; COHRA, Center for Oral Health Research in Appalachia; SNP, single nucleotide polymorphism; y, year; *TAS1R2*, taste 1 receptor member 2; *TAS1R3*, taste 1 receptor member 3; *GNAT3*, G protein subunit alpha transducin 3; *PCSK1*, proprotein convertase subtilisin/kexin type 1; *INSR*, insulin receptor.

As seen in Fig. 1, variation in the brain reward system may impact the hypothalamic control of downstream appetite and T2D pathways. The solute carrier family 2 (*SLC2A2*) gene encodes GLUT2, a facilitated glucose transporter, which has both transport and receptor functions. Due to its low affinity for glucose, GLUT2 has been proposed to be a glucose sensor involved in regulating blood glucose concentration. *DRD2* encodes dopamine receptor D2 and the binding of dopaminergic agents such as bromocriptine to dopamine receptors is used to treat T2D symptoms including hyperglycemia, insulin resistance, free fatty acids, and TG [62]. Variation in sweet taste perception may impact dietary sugar intake and CMR. *TAS1R2* and *TAS1R3* code for proteins (T1R2 and T1R3) that heterodimerize to form sweet taste receptors. *GNAT*3 encodes the alpha subunit of gustducin, the G protein responsible for transmitting sweet, bitter and umami tastes from taste receptors. In preschool-aged children, SNP variants in *TAS1R2* have been associated with dietary sugar intake [45, 46] and SNP variants in *GNAT3* have been nominally associated with dental caries [47]. Bidirectional relationships between sweet taste perception and response with the endocannabinoid system have been identified [62, 63]. Endocannabinoids bind with G-protein coupled cannabinoid receptors, i.e., CB1 and CB2 encoded by *CNR1* and *CNR2*, respectively [64]. In mice, administration of cannabinoids enhanced sweet gustatory nerve response and increased sugar seeking behaviour via CB1 receptors, which are concurrently expressed with T1R3 in taste cells [62, 64]. Variation in insulin-mediated glucose transport may impact CMR. Proprotein convertase subtilisin/kexin type 1 and 2 (*PCSK1*, *PCSK2*) encode proprotein convertase 1/3 and 2 (PC1/3, PC2) respectively. These enzymes are involved in the cleavage of proinsulin to insulin and proglucagon to glucagon. A recent study demonstrated that the PC1/3 gene processes proinsulin in healthy human β-cells while the PC2 gene was only detectable in β-cells from donors with T2D [65]. Therefore, variation in PC1/3 may negatively impact regular insulin processing. *INSR* encodes preprotein precursors cleaved into alpha and beta subunits of the heterotetrameric insulin receptor and SNP variants in *INSR* have been associated with CMR factors in preschool-aged children [48]. Binding of ligands including insulin and insulin like growth factor 1 (IGF-1) along with insulin receptor substrate 1 (IRS1) or insulin receptor substrate 2 (IRS2) to the insulin receptor results in activation of the insulin signalling pathway. Under basal conditions the GLUT4 receptor is sequestered in GLUT 4 storage vesicles and released to the plasma membrane in response to elevated blood sugar (insulin-dependent) or increased energy needs [66, 67].

### 2.12 Gaps in Research Investigating the Impact of Genetics on Sugar Intake and Cardiometabolic Risk

Preschool-aged children are underrepresented in genetic research. However, transitioning from candidate gene approaches to GWAS and epigenetic methods will likely expedite the discovery of genetic patterns underlying CMR as larger samples become available in this population. Healthful habits introduced by families may be able to reprogram epigenetics and establish healthy lives for children, but the actual epigenetic effect of excess sugar intake remains unclear. SSB intake is associated with adiposity and evidence suggests that hypermethylation of peroxisome proliferator-activated receptor Alpha (*PPARα*) and *CPT1A* by fructose consumption is one mechanism driving this effect [68]. The specifics of this pathway have not been elucidated and other genetic pathways by which fructose influences adiposity may exist. Future research should continue to investigate the biological basis of SNP associations with dietary sugar intake and CMR in young children as well as how dietary sugar intake impacts epigenetic programming of CMR over a lifespan.

## 3. Methodological Considerations: Investigating the Relationship between Dietary Sugars, Genetics, and Cardiometabolic Health in Preschool-Aged Children.

### 3.1 Assessing Dietary Sugar: Inconsistencies in Measuring Added and Free Sugar Intake

Monitoring dietary sugar consumption in preschool-aged children is essential to assess adherence to national sugar reduction guidelines. Given the varying definitions and descriptions of free and added sugars in the literature, together with the lack of consensus of recommended daily intakes globally, evaluations of both added and free sugar intakes are challenging. Currently, there are various methods used to determine the amount of total sugars present in foods or in the diet of preschool-aged children. Some approaches commonly used to evaluate EI include food frequency questionnaires, food records, direct observation, dietary screening questionnaires, 24-hour dietary recalls and the doubly labeled water method [69, 70]. However, assessing added or free sugar intake is especially challenging because there are limited nutrient databases that include added or free sugar content of foods. Furthermore, there is no universal system that is used in research to determine added sugar intake since its definition varies. Nonetheless, there are some existing approaches in the literature that may be used for estimating total and added sugar intake in preschool-aged children. In particular, the four methodologies that will be examined in this review include, the Automated Self-Administered 24-hour Recall (ASA24®), Nutrition Data System for Research (NDSR), ESHA’s Food Processor® Nutrition Analysis Software, version 11.11 (ESHA Research, Salem, OR, USA) and the disaggregation method. The ASA24, NDSR and ESHA are commonly used methodologies, whereas the disaggregation method is a newer approach used to assess added sugar intake. An overview of the strengths and limitations of these methodologies in estimating sugar intake is provided in Table 6 (Ref. [71, 72, 73, 74, 75, 76]).

**Table 6.** **Summary of strengths and limitations of methodologies by which sugar intake is assessed**.

	Strengths	Limitations	Reference
ASA24	∙ Cost and user-friendly	∙ May require regional adaptions depending on varying added sugar definitions between jurisdictions	[71]
	∙ Multiple pass method helps enquire details regarding forgotten foods, food source, preparation methods and additions to foods	∙ Recall bias	
	∙ Can be used across various population subgroups, including preschool-aged children	∙ Time-consuming for participant to complete, especially for multiple day diet recalls, as one 24-hour food record takes approximately 30 minutes to complete	
	∙ Can be adapted for use across different countries and regions		
	∙ Digital images may help with accurate portion size reporting		
	∙ Useful for assessing dietary intake and measuring added and total sugar intakes		
NDSR	∙ Updated annually	∙ Requires license and fee	[72, 73, 74]
	∙ NCC database provides a greater number of nutrient and food components compared to ASA24 and ESHA	∙ Designed for US population and may not be suitable across different countries due to differences in added sugar definitions and formulations of food products	
	∙ NCC database provides a greater number of food brands and items compared to ASA24 and ESHA	∙ Recall bias	
	∙ Readily outputs added sugar values	∙ Interviewer bias	
	∙ Measures sugars in two ways: carbohydrates and caloric sweeteners		
ESHA	∙ Widely used in the literature	∙ Requires license and fee	[75]
	∙ Recall bias is not a major concern	∙ Manual inputting is required for food items within a diet record that are not present in the ESHA databases	
	∙ Includes complete information from product nutrition labels, including brand-name products and restaurant menu items	∙ Nutrient profiles generated are not as extensive compared to ASA24 and NDSR databases	
	∙ Suitable for inputting recipes	∙ Only limited types of foods have been populated with added sugar data	
		∙ Added sugar content of mixed foods is determined manually	
Disaggregation Method	∙ Cost-friendly	∙ May require subjective decision-making by the user (bias)	[76]
	∙ Flexible for use across jurisdictions that have different definitions for added sugars	∙ Accuracy of the total and added sugar values assigned to the ingredients depends on the quality and accuracy of the food composition database	
	∙ Can be used to assess free sugars	∙ Requires detailed recipes for the food items	
		∙ May be time-consuming for researcher	

#### 3.1.1 Automated Self-Administered 24-Hour Recall (ASA24®) Dietary Assessment Tool 

The ASA24, developed by the National Cancer Institute, is a self-administered, web-based dietary assessment tool available for no cost to researchers, instructors, and clinicians [77]. Individual single or multiple 24-hour food recalls are automatically coded to generate nutrient information from all foods reported by the respondents. US versions of the ASA24 use the Food and Nutrient Database for Dietary Studies (FNDDS) [77]. The ASA24 is based on the Automated Multiple Pass Method, a previously validated tool used to accurately assess mean total energy intakes [77, 78]. This multiple pass approach is effective in probing respondents for additional details regarding the food source, preparation methods, portion sizes, additions to foods, forgotten foods or beverages [77]. This approach has been previously shown to reduce bias and may ultimately provide the researchers with more accurate estimates of added and total sugar intakes [69]. A previous validation study of the ASA24-Canada conducted by our team found that parental reporting aligns relatively closely with the observed EI of preschool-aged children, making it a suitable tool for measuring sugar intake in this group [70]. Additionally, the ASA24 has also been validated against intakes of added sugar, where respondents accurately reported their added sugar consumption and did not underreport their intake of sugary snacks when using the ASA24 [69, 79]. An additional feature of the ASA24 is its ability to output the required variables for calculating Healthy Eating Index (HEI) scores, which is a measure of diet quality in compliance with US dietary guidelines [77]. Hence, the ASA24 can also be used to measure diet quality of preschool-aged children via HEI scores. Despite these benefits, the ASA24 has limitations that also need to be considered for data review and interpretation. One concern is that the added sugar definition used by the ASA24 database may not be universally relevant for use. The US version of ASA24 uses nutrient data derived from the Food Patterns Equivalents Database (FPED) and FNDDS maintained by the USDA Food Surveys Research Group at the Beltsville Human Nutrition Research Center [71]. The ASA24 added sugar component is derived from FPED, which follows the US FDA added sugars definition, while the ASA24 total sugars component is derived from FNDDS [72, 80]. Therefore, ASA24 requires further regional adaptions if used outside of the US. Although the ASA24 nutrient database has been adapted for the Canadian and Australian contexts using the Canadian Nutrient File (2015) and Australian Food, Supplement and Nutrient Database (2011-13), respectively, further adaption is warranted for use in other countries [77]. In particular, the FPED measures added sugar contents in foods by first, identifying food ingredients containing added sugars and second, calculating the added sugar content per 100 grams of the ingredients and food items [80, 81]. For more complex foods, recipes are required to calculate the added sugar contents of the ingredients [80, 81]. In addition, the FPED assumes that 100 grams of fruit nectars and fruit juice contain 5.4 grams and 2 grams of naturally occurring sugar, respectively, values which are subsequently subtracted from total sugars to obtain an added sugar value. The FPED also derives added sugar values by comparing the difference in total sugars for the same food in its unsweetened and sweetened forms (i.e., canned pineapple packed in water versus syrup) [80]. Of note, foods and beverages not readily present in the FNDDS and FPED databases will require further manual entry in the ASA24, which can introduce human error and potentially misreport the total and added or free sugar contents of these items. When reporting a meal, the food or drink items the respondent cannot find are identified as “unfound food” by ASA24. The respondent is later prompted to answer a series of general follow-up questions about the unfound food items for better identification, which are used to assign a default food code based on the closest match. However, discrepancies may exist between the nutrient profile of the actual item consumed compared to the generated ASA24 food description for select unfound food items. For example, “natural peanut butter” does not have a unique food code in ASA24 and is therefore, considered an unfound food. Based on the additional details provided by the respondent, ASA24 may default to code natural peanut butter as “Peanut butter, smooth type, fat, sugar and salt added”, thus generating a nutrient profile with greater added sugar content compared to true intake. However, the ASA24 includes over 10,000 images of food and beverage items to assist respondents in accurately estimating their food intake for the majority of foods. Another consideration is that the differing databases for added versus total sugars result in inconsistent estimates when comparing across these two values of a particular food. For example, after converting estimated added sugars to grams, added sugar values may incorrectly exceed the total sugars present in a particular food, as ASA24 reports total sugar and added sugars from FNDDS and FPED, respectively and without adjustment. Furthermore, converting added sugars to energy (kcal/gram) may result in energy from added sugar values exceeding the FNDDS variable kcal (total energy). Also, when using the ASA24, obtaining accurate estimates of dietary sugar intakes in preschool-aged children depends on the parent’s ability to recall correctly and their level of computer literacy. Nonetheless, the ASA24 provides a feasible alternative to interview-administered 24-hour recalls and is an effective tool to assess sugar intake in preschool-aged children.

#### 3.1.2 Nutrition Data System for Research (NDSR)

The University of Minnesota Nutrition Coordinating Center’s (NCC) NDSR software is another commonly used 24-hour recall system for dietary analysis and can also readily measure added sugar contents of foods and beverages. This PC-based software requires a license and has a cost associated with its use and training. Depending on the type of license, the price of purchasing and installing an initial copy of the NDSR can range, with additional costs for training and certification. The NDSR software is supported by the proprietary NCC Food and Nutrient Database, and both are updated annually to capture changes in the marketplace and food supply as well as, to introduce new analytic and diagnostic data [73]. The interviewer-administered approach of the NDSR eliminates the need for the parents to have internet access or computer literacy, as well as reduces the risk of uninformed classification of their child’s diet due to guidance from the interviewer who has knowledge of food codes. In addition to the ASA24, NDSR is one of the few nutrient databases with data on added sugar content of food products. However, the NDSR may hold an advantage over the ASA24, since it contains a more extensive list of nutrients, food products and brands [73]. The NDSR calculates added sugars in two different ways, namely by total sugar and by available carbohydrates centered on the chemical structure of the carbohydrates in various types of caloric sweeteners [73]. The calculation of added sugars by available carbohydrates for single ingredient foods (e.g., sugar, honey, molasses, syrups, etc.) involves the NCC database to assign an added sugar value that is equivalent to the available carbohydrate value of the particular item [74]. Therefore, added sugars by available carbohydrates contain all carbohydrates that are produced commercially and added to sweeten food products or beverages, including monosaccharides, disaccharides, oligosaccharides and polysaccharides. As a result, the added sugar value (by available carbohydrates) may exceed the amount of total sugars (g) for the same food. In contrast, the added sugars by total sugars value assigned by the NCC database for these single ingredients foods is equivalent to the total sugar value of the food item. The added sugar by total sugar value only includes monosaccharides and disaccharides that are added as caloric sweeteners to foods and beverages. For all other foods in the database, the added sugar value using either method (by available carbohydrates and by total sugars) is extracted based on the total amount of these single ingredient foods present in a given recipe or product formulation [74]. Although the exact method by which NDSR calculates added sugars is not known, it has been previously speculated that NDSR likely uses an equation that considers the products ingredient list to help determine and calculate the amount of added sugar present in the food [82]. The NDSR also has drawbacks including the potential of interviewer bias, cost limitations and recall bias. Another important factor to consider is that of the few databases that output added sugar information, not all of them produce consistent values that agree across different databases. A study comparing the NDSR database and USDA’s FPED used by ASA24, found that while the total sugar content was the same, there were discrepancies in the added sugar values of foods [82]. For example, the added sugar values for cakes, brownies, donuts and sweetened applesauce were greater using the NDSR compared to the USDA database. Whereas items including vanilla flavoured yogurt, crackers, cookies and canned fruits in heavy syrup provided greater added sugar values using the USDA database [82]. Hence, the definition and approach of calculating added sugars in foods varies significantly between databases. Another limitation of the NDSR is that it is based on the nutrient composition values for the US food supply [73]. Therefore, the NDSR may not be suitable for use outside of the US, due to varying food preparation and fortification methods across different countries. Nonetheless, the NDSR may provide a comprehensive understanding of the added sugar content in foods through its various nutrient outputs in preschool-aged children.

#### 3.1.3 ESHA’s Food Processor® Nutrition Analysis Software

ESHA’s Food Processor Program is another method for obtaining food composition values for foods and beverages and may be used to collect added sugar intakes in preschool-aged children. Similar to the NDSR, ESHA’s Food Processor requires purchase of a license [75]. However, unlike the ASA24 and NDSR, ESHA is not a 24-hour recall dietary assessment tool, but rather a nutritional analysis software in which diet records are collected and then coded into the software by trained personnel [83]. ESHA may be a useful tool for observational research studies and randomized controlled trials that include dietary assessment. The databases sourced by ESHA are widely used in the literature and may provide high quality energy and nutrient intake data [84]. ESHA’s Food Processor software uses the Food and Nutrition Database that compiles nutrient profile and composition data from the USDA’s FNDDS and other databases and includes information regarding many brand-named food products and restaurant menu items [75, 83, 85]. In comparison to the USDA’s FNDDS and NDSR’s NCC databases, the ESHA database does not contain as extensive nutrient profiles; however, it does include information from product nutrition labels for dietary analysis. An additional feature of ESHA is that it can be used to create recipes and hence, the data can be tailored to the user’s needs. Similar to the ASA24 and NDSR, the ESHA Food Processor may be used to estimate the total and added sugar contents of foods in preschool-aged children. After the FDA announced labeling changes, specifically, for US nutrition labels to include added sugar content, ESHA began populating its databases with added sugar content of food products according to the FDA’s added sugar definition. Reportedly, ESHA has implemented added sugar data for approximately 12,000 food products within its databases [75]. However, the ESHA Food Processor is still largely lacking in this regard, as only limited types of foods have been populated with added sugar data. In particular, ESHA only calculates added sugar data for foods that do not contain any sugar (thus, if total sugar equals 0, then added sugar equals 0), for whole foods that only contain natural sugar (added sugar equals 0), and for single ingredient foods like sugar and honey (added sugar equals total sugar) [75]. Likewise, ESHA populates added sugar data for mixed foods if the food manufacturers have indicated an added sugar value [75]. For foods that do not fall within these categories, the added sugar component will remain blank [75]. As such, researchers using the ESHA Food Processor will have to determine added sugar content of mixed foods manually. This process is time-consuming, requiring a detailed inspection of the food record to correctly identify food products that may potentially contain added sugars. Although ESHA has expanded its nutrition databases to include and automatically generate added sugar information for certain foods, there remains key gaps in obtaining accurate estimates of sugar intake data.

#### 3.1.4 Disaggregation Method

**Fig. 2.**
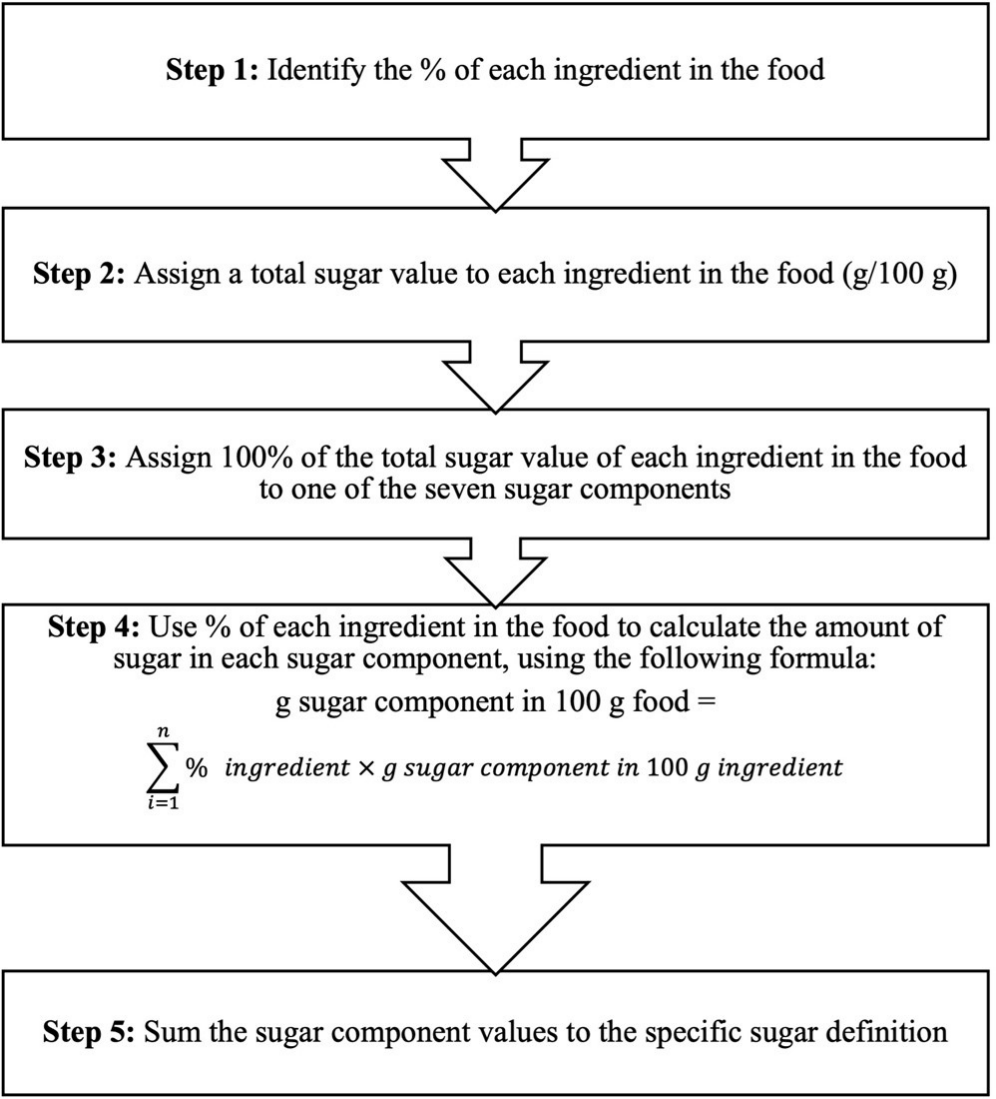
**Five steps for estimating the added or free sugar contents in food products according to the disaggregation method**. Figure is reproduced with permission from, [76].

Introduced by Amoutzopoulos *et al*. [76] the disaggregation method is a novel UK-based system designed to measure dietary intake of added and free sugars and can also be applied to preschool-aged children. Unlike the above approaches, a unique and important feature of this method is that it can be adapted to different sugar definitions across jurisdictions and is responsive to changes in definitions [76]. The disaggregation method pre-assigns total sugars into seven sub-categories including table sugar, sugar-based sweeteners, honey, fruit and vegetable juice, fruit puree, stewed fruit and dried fruit [76]. The summation of these individual ‘sugar components’ are subsequently used to estimate the added sugar contents of the food item according to the different sugar definitions [76]. The disaggregation method involves five steps, as illustrated in Fig. 2 (Ref. [76]).

First, the proportion of each ingredient present in a food item is identified from the food label. Second, a total sugar value, obtained from a food composition database, is assigned to each ingredient per 100 g. Next, 100% of the total sugar value of each ingredient is assigned to one of the above seven sugar components. Following this third step, using the predetermined formula (Fig. 2), the proportion of each ingredient in the food item is used to calculate the amount of sugar for each sugar component. Finally, these sugar component values (in 100 g of foods) are summed according to the specific sugar definition, to provide an estimate of the added or free sugar content of 100 g of food.

The step-by-step breakdown of the disaggregation method illustrates the flexibility in this approach in determining the added sugar contents of food products for different sugar definitions (i.e., added and free sugars). However, this system may introduce bias, as users are required to subjectively decide which sugar component is appropriate for the food product and accordingly, assign the sugar value. Furthermore, the accuracy of the total and added sugar values assigned to the ingredients are dependent on the quality of the food composition database. Another limitation is that users are required to have detailed information regarding the recipes for the food items, along with the total sugar data, which may not be feasible in some situations. Further, the calculated added sugar value of a particular food item may not be consistent across jurisdictions, due to the differing sugar definitions and fortification methods, and therefore, would necessitate recalculation. Despite these drawbacks, the disaggregation method can be adapted using country-specific food composition databases and therefore, flexibly applied to different definitions of added and free sugars. This method may also be used to assess intake of different populations, including preschool-aged children.

#### 3.1.5 Gaps in Dietary Assessment Tools for the Estimation of Dietary Added and Free Sugars

Although many methodologies exist for dietary assessment and nutrient analysis, accurately determining the contents of dietary added and free sugars remains a challenge. Presently, very few food composition databases comprehensively include added and free sugar data. Of the limited nutrient databases that include this information, it remains unclear how exactly these sugar values are generated and whether this data can be used across jurisdictions that have different product formulations. Since there is no universal system for determining added sugar, researchers may be presented with a challenge when choosing between different databases, as the methods of estimation may yield varying added sugar values. Furthermore, the chosen database may produce diverse results compared to other databases, which may influence the perceived quality of the diet. Also, since the validity of dietary assessments produced by different software depends mainly on the quality of the nutrient and food composition data, regular updates of these systems are necessary to capture any changes in food product formulations and sugar definitions. Although these systems have potential to generate high quality data, there are multiple trade-offs to consider when choosing between dietary assessment tools and a degree of subjectivity is present in all four methods. Despite these limitations, all four methodologies reviewed can be implemented for estimating sugar intakes in preschool-aged children. The following section will discuss methodological considerations when investigating dietary sugar intake through genetic lenses.

### 3.2 Genetic Research Methodologies 

#### 3.2.1 Controlling for Covariates

Covariates including ethnicity and genetic variants with established impact on CMR should be considered and controlled for in the study design or during statistical analysis. Failure to consider allelic heterogeneity (ethnic specific variation in allele frequency or differences in the allele that confers risk) may introduce artificial associations due to population stratification [86]. Furthermore, genetic variants with established impact on CMR should be controlled for when investigating the impact of novel gene variants. Controlling for known variants will help improve detection of novel and independent SNP associations.

#### 3.2.2 Individual Risk Variants, Combined Risk Variants, and Genetic Risk Score 

Identifying gene variants of interest has historically followed a targeted gene approach starting with a scientific hypothesis based on known or proposed biological pathways. However, this approach will likely be replaced as GWAS becomes more affordable and data from increasingly larger samples become available. Outcomes of large GWAS projects include the generation of thousands of risk loci of small effect size, loci that have not reached genome-wide statistical significance in their association with CMR, and the need for careful interpretation of data. While stringent p-value thresholds and Bonferroni correction factors help to avoid false positives, these tests can also introduce false negatives. Of note, risk variants with small effect sizes and no significant impact on CMR alone may have a large effect together. In two cohorts of 174 and 165 obese Caucasian children, children with both risk variants IRS1 Arg 972Gly and IRS2 Asp1057Gly had a 25 to 35% decrease in insulin sensitivity compared to children with no risk alleles [60]. Conversely, children with either risk allele alone only showed slightly reduced insulin sensitivity. These findings demonstrate the importance of considering complementary roles that genes may have on particular cardiometabolic pathways. Genetic risk scores (GRS), estimates of the cumulative contribution of genetic factors to a specific outcome in an individual, are often adopted in response to the limitations of small effect sizes of individual gene variants. They are commonly calculated by multiplying the number of risk alleles by beta (effect size) or by relative risk for binary (affected/unaffected) outcomes, and summing products across SNPs [87]. The score is then used as a risk factor and tested for its ability to predict a quantitative variable or an outcome in an independent sample. GRSs have two main purposes: (1) Predict the likelihood of an individual developing a particular outcome. (2) Estimate the level of predictive power that is captured by associated variants. Additionally, GRS can incorporate multiple factors including genetic data, environmental, phenotypic, and/or demographic information. One important consideration when interpreting GRS is that most GWAS have been completed on individuals of European descent and allelic heterogeneity should be considered when interpreting and planning GRS in mixed ethnicities.

#### 3.2.3 Methodologies Investigating Epigenetic Interactions

High fructose and SSB intake may induce epigenetic changes and influence methylation patterns in tissue and time specific manners, eventually increasing CMR [68]. However, epigenetic analysis in young children and adults remains sparce due to the invasive nature of sample collection. Most work in this field uses adult blood and saliva samples, which does not reflect the complexity of tissue-specific epigenetic effects [88]. Still, pathways relevant to taste, food preference, and CMR are present in these two tissues and epigenetic investigation into these pathways may yet yield valuable insight. A review of the ethical, legal, and social challenges of epigenetic testing in children has been previously published [88].

### 3.3 Cardiometabolic Research Methodologies

Measuring meaningful CMR in preschool-aged children is an important and challenging task, which may track into adulthood [89]. Measurements such as anthropometrics, insulin resistance, BP, and dyslipidemia are used to assess pediatric CMR. Anthropometrics including body weight, WC, and BMI are the most investigated CMR factors in preschool-aged children as these variables are non-invasive and relatively simple and inexpensive to collect. A strong link between obesity and CMR, e.g., insulin resistance is well established in pediatric populations [90]. Furthermore, given that visceral adipose tissue (VAT) is well known to have a greater impact on metabolic dysregulation and controversial evidence of metabolically healthy obese children, VAT-specific adiposity measurements such as WC may be preferrable [91]. There is also evidence for insulin resistance in children, however, the lack of accepted reference ranges is a barrier to the interpretation of these findings [90] A recent review addresses the validity of current methods of measurement and diagnosis of pediatric insulin resistance [92].

## 4. Gaps in the Research and Recommendations

There is emerging evidence linking added and free sugar intakes with CMR factors in preschool-aged children, but further research, especially longitudinal follow-up, is needed. Research in this area becomes crucial when providing guidance for early life interventions for young children and requires consistency, specificity, and consideration of what it means to work with this unique and sparsely investigated population.

To ensure collection of accurate dietary sugar estimates, which can be compared with data from various jurisdictions, researchers should use consistent sugar definitions and select dietary collection tools specific to each study’s aim, design, and sugar definition. They should also limit subjectivity of dietary assessment tools and formulate correction equations for existing sugar biomarkers that should be used consistently. Flexible approaches, such as the disaggregation method can help compare data reported by sugar reduction programs and dietary guidelines, which use varying definitions of sugar. As well, newer dietary systems that use a uniform added sugar definition relevant between jurisdictions (i.e., free sugar) are required, or alternatively, should be flexible in their approach to account for multiple definitions. These approaches will help increase the accuracy and objectivity of measurements used to estimate free and added sugar content in food products.

Preschool-aged children comprise a relatively healthy population, whose CMR markers are rarely acute. Since CMR factors are poorly defined in this population, research should use non-invasive anthropometric measurements such as WC, body weight, and BMI as a first choice until the strengths and limitations of available measurements are clarified. Establishing reference ranges for insulin resistance in children may greatly improve analyses in this field.

Poorly defined CMR factors in combination with the fact that the contribution of genetics to dietary sugar intake is likely polygenic, makes it difficult to form conclusions from the sparse research available. Genetic factors are revealing themselves to play increasingly important roles in the pathogenesis of cardiometabolic disease in young children. A growing number of candidate-gene studies have provided important insights into potential new lines of investigations. For example, genetic variants involved in sweet taste and sugar metabolism may be associated with dietary sugar intake and CMR in preschool-aged children [45, 46, 48]. Given the polygenic nature of obesity, future research should consider how risk alleles with complementary roles may negatively impact children’s health using methods such as GRS and GWAS.

Despite the challenges involved in working with young children, further research on sugar intake and CMR factors is warranted to inform policy decisions and guidelines for healthy eating in children. Taken together, these recommendations will contribute to a robust research body and help in the development and implementation of more effective public health policies and guidelines aimed at reducing sugar intakes and CMR in preschool-aged children.

## 5. Conclusions

In conclusion, this review highlights the need for further longitudinal research on sugar intake and cardiometabolic risk factors in preschool-aged children, while also providing key recommendations for researchers to improve consistency in sugar definitions, select appropriate dietary collection tools, limit subjectivity in assessment tools, and consider non-invasive biomarkers of cardiometabolic disease. The review also emphasizes the importance of accounting for the polygenic nature of cardiometabolic disease in future research, using approaches such as genome risk scores and genome-wide association studies. Following these recommendations will support a robust research body to help inform policy decisions and healthy eating guidelines designed to reduce cardiometabolic risk in young children.
